# Reversal of Rocuronium-Induced Neuromuscular Blockade by Sugammadex Under Sevoflurane and Desflurane Anesthesia in Children

**DOI:** 10.5152/eurasianjmed.2022.22090

**Published:** 2023-10-01

**Authors:** Gamze Pirinç Şaşıoğlu, Pınar Kendigelen, Ayşe Çiğdem Tütüncü, Güner Kaya

**Affiliations:** Department of Anesthesiology and Intensive Care, Istanbul University-Cerrahpaşa, Cerrahpaşa Medical Faculty, Istanbul, Turkey

**Keywords:** Pediatric anesthesiology, desflurane, sevoflurane, rocuronium, sugammadex

## Abstract

**Objective:**

In children, neuromuscular blockers may have different effects with various inhalation agents and incomplete reversal of the blockade continues to be a problem. The aim of the study is to observe the effects of sugammadex on reversing the blockade by a single dose of rocuronium, the peak inspiratory pressure, hemodynamic parameters in children under sevoflurane and desflurane anesthesia.

**Materials and Methods:**

One hundred forty-eight children aged 2-10 years old, to be operated under short-term general anesthesia, were enrolled in this prospective randomized controlled trial. After induction, the patients were intubated at the end of TOF (Train-of-four) ratio of 1.0-0. The time taken until TOF reached 0.25 was recorded, and 2 mg/kg sugammadex was administered to the patients. The period until the TOF was at least 0.9 and in the first 10 minutes after sugammadex injection, peak inspiratory pressure, the systolic-diastolic arterial pressure, and the heart rate wers3e monitored and possible side effects were observed in the recovery room.

**Results:**

Following the injection of sugammadex, the time taken for TOF of 0.25 to reach **>**0.9 was significantly shorter in the 2-4-year-old age group under sevoflurane anesthesia. After sugammadex injection, a small but statistically significant increase in peak inspiratory pressure values was observed in Group D at the 2nd, 5th, and 10th minutes.

**Conclusion:**

Rapid and complete recovery was achieved from the block induced by a single dose of 0.6 mg/kg rocuronium by the use of sugammadex which did not give rise to any side effects.

**Main Points**

- Sugammadex reverses neuromuscular blockade by encapsulating and binding of non-depolarizing muscular blockers.
- The development of the neuromuscular junction is completed by 2 years of age. However, there may still be differences in the neuromuscular junction up to 4 years of age compared to adults.
- The faster increase in sugammadex concentration in the neuromuscular junction results in faster recovery. As age decreases, the block reversal therefore may get faster.
- Whether desflurane increases airway irritation and resistance and whether its use with sugammadex would increase these adverse side effect incidences will require further case reports.
- Further research is needed about the use of sugammadex in order to determine its side effects and the safety of combination with other agents.

## Introduction

Sugammadex shows the reversal of neuromuscular blockade effects without increase in the cholinergic activity, unlike the classical anticholinesterase inhibitors, reverses neuromuscular blockade by encapsulating and binding of non-depolarizing muscular blockers. It is used only as the reverse of the aminosteroidal neuromuscular blocking drugs rocuronium and vecuronium.^1^ The approval of its use only in children above the age of 2 years limits the availability of data in the literature on prospective randomized trials. However, its usage in differing and specific conditions led to an increased number of case reports.^1^

Given their immaturity and the inadequacy of the elimination functions, the neuromuscular synapses in infants and small children are sensitive to blocker agents.^2^ Postoperative residual muscular blockade can facilitate pulmonary and respiratory complications. The complete reversal of neuromuscular block should be the main target in every case in order to decrease these risks.

The primary aim of this study is to observe the effectiveness of sugammadex and Train-of-four (TOF) in the reversal of the intubation dose of rocuronium and evaluation of the resulting airway pressures in 2-10-year-old children placed under short-term anesthesia with sevoflurane or desflurane. The secondary aim includes whether the sugammadex effect differed under sevoflurane and desflurane anesthesia and to observe the possible side effects of sugammadex. The patients of 2-4 and 5-10 years of age were placed in 2 subgroups in order to determine the age dependence of the effects evaluated.

## Materials and Methods

Before patient enrollment, this prospective randomized controlled study was approved by the Institutional Ethics Committee documented as No: 83045809-604.01 and registered at clinicaltrials.gov (NCT03795259). Children aged 2-10 years old with ASA I-II and to be operated under short-term general anesthesia for elective lower urinary system (cystoscopy, circumcision, etc.) or lower abdominal surgery (inguinal hernia, etc.) were enrolled in the study after the written informed consent of the parents. Children with organ failure, upper airway infection, asthma, exposure to secondhand smoke, muscular diseases (myasthenia gravis, muscular dystrophies, etc.), obesity (body mass index (BMI) > 30%), allergy to rocuronium or other medications, suspected malignant hyperthermia, using calcium-channel blocker, and those children likely to use sugammadex within the 3 days before the surgery of this study, or whose families refused to sign the informed written consent form were excluded from the study. In addition, patients with hemodynamic instability and hemorrhage within per-operative period, prolonged surgery duration, and in need of additional dose of rocuronium were also excluded from the study.

The primary aim of the study was to determine the mean reversal time of muscular blockade after sugammadex in the 2 anesthesia groups. The minimal number of participants needed for the study was calculated on the formula n = [*Zα* **/**E]^2^. A pilot study with a total of 10 patients was carried out to determine the TOF-T_S_ mean **± **SD, where the *α* and the power were determined, respectively, as 0.05 and 0.85, as a result that minimally 72 participants were needed per anesthesia group or a total of 144 for the study. Considering the possibility of excluding patients from the study, we planned to include a total of 160 participants with 2 subgroups which are group S for sevoflurane and group D for desflurane. Each group had 80 participants.

The inhalation anesthetic agents were assigned to the participants by draw and the measurements were made by an anesthesiologist who was blinded to the agent used. Preoperatively, the children were administered intravenous (i.v.) midazolam (0.05 mg/kg) (Zolamid, Defarma-Turkey) and ketamine (0.5 mg/kg) (Ketalar, Pfizer-USA). The standard monitorization technique determined by the ASA was implemented. As the patients were placed on the surgery table, fan heaters were started to maintain body temperature ≥ 36˚C and followed up by tympanic membrane thermometer in the operation theater to prevent hypothermia. Anesthesia was induced by pentothal (5 mg/kg) and fentanyl (2 μg**/**kg) i.v. For intraoperative acceleromyography monitoring (TOF-Watch®SX Organon-Ireland), the *nervus ulnaris* and *musculus adductor longus muscles *were selected on the arm without the noninvasive blood pressure cuff. For increasing the neuromuscular response stabilization, a 1-Hz pulse for 1 minute was applied to the ulnar nerve of the wrist. It was subsequently stimulated every 15 seconds with 0.2 millisecond square wave stimuli of 2 Hz at the TOF mode and ipsilateral adductor pollicis muscle contractions were measured. After stabilization of the response to TOF, the internal calibration function of the acceleromyography was completed. After observing a stable baseline for at least 2 minutes, the patients were administered 0.6 mg/kg i.v. rocuronium (Esmeron, MSD-Germany). The time of completion of the i.v. injection was taken as 0 minute, and TOF measurements were made every 15 seconds.

When the time to TOF 0 was reached, the time for rocuronium to take effect, and the patients were orotracheally intubated. Next, in addition to air-oxygen mixture, the inhalation agent was set to 1 MAC. Using the “Datex Ohmeda S/5 Avance” anesthesia device in the volume-controlled mode, the intraoperative mechanical ventilation settings were adjusted to FiO_2_: 40%, I/E: 1/1.5 or 1/2, PEEP: 5 cmH_2_O, 8 mL/kg tidal volume and with the respiratory rate meeting the end-tidal CO_2 _level of 35-40 mmHg. TOF measurements were recorded every 5 minutes during the first 15 minutes and thereafter, continuously, every 15 seconds until the rocuronium effect duration reached to TOF of 0.25. At this stage, while patients were still under inhalation anesthetics, sugammadex (2 mg/kg) (Bridion, MSD-USA) was administered and TOF levels were kept measured (sugammadex was prepared by diluting the dose with 0.9% NaCl to 10 mg/mL in order to increase the accuracy of dosing in the children and was given within 30 seconds). The time taken from TOF 0.25 to ≥ 0.9 was recorded as *T*_S_, the recovery time from neuromuscular block. The time before sugammadex injection was taken as 0 minute, and the peak inspiratory pressure (PIP), the systolic-diastolic arterial pressure (SAP-DAP), and the heart rate (HR) were recorded at 0, 1, 5, and 10 minutes. The observation for sugammadex side effects was maintained during these 10 minutes. Acceleromyography was terminated when reaching TOF ≥ 0.9. The inhaled anesthetic use was continued until the surgical procedure was terminated, and the patients were extubated with the reversal of the muscular block. Postoperative analgesia was implemented according to the patient’s need and follow-up for possible side effects was continued to be observed for 30 minutes in the recovery room. Throughout the operations, the SAP-DAP, HR, and PIP were recorded every 5 minutes.

### Statistical Analysis

The Statistical Package of the Social Sciences version 15.0 (SPSS Inc.; Chicago, IL, USA). software for Windows program was used for data analysis. Descriptive and categorical variables were expressed numerically and in percentages. The numerical variables were expressed in terms of the mean, standard deviation, the median, and the minimum-maximum values. As the numerical variables did not meet the assumptions of normal distribution, the Mann-Whitney *U*-test was used for comparison of 2 independent groups. The differences of the dependent groups were analyzed with the Wilcoxon test and interpreted after the Bonferroni correction. The chi-square test was used to compare data ratios in the groups and *P *< .05 was accepted to represent statistical significance.

## Results

**Figure 1.**
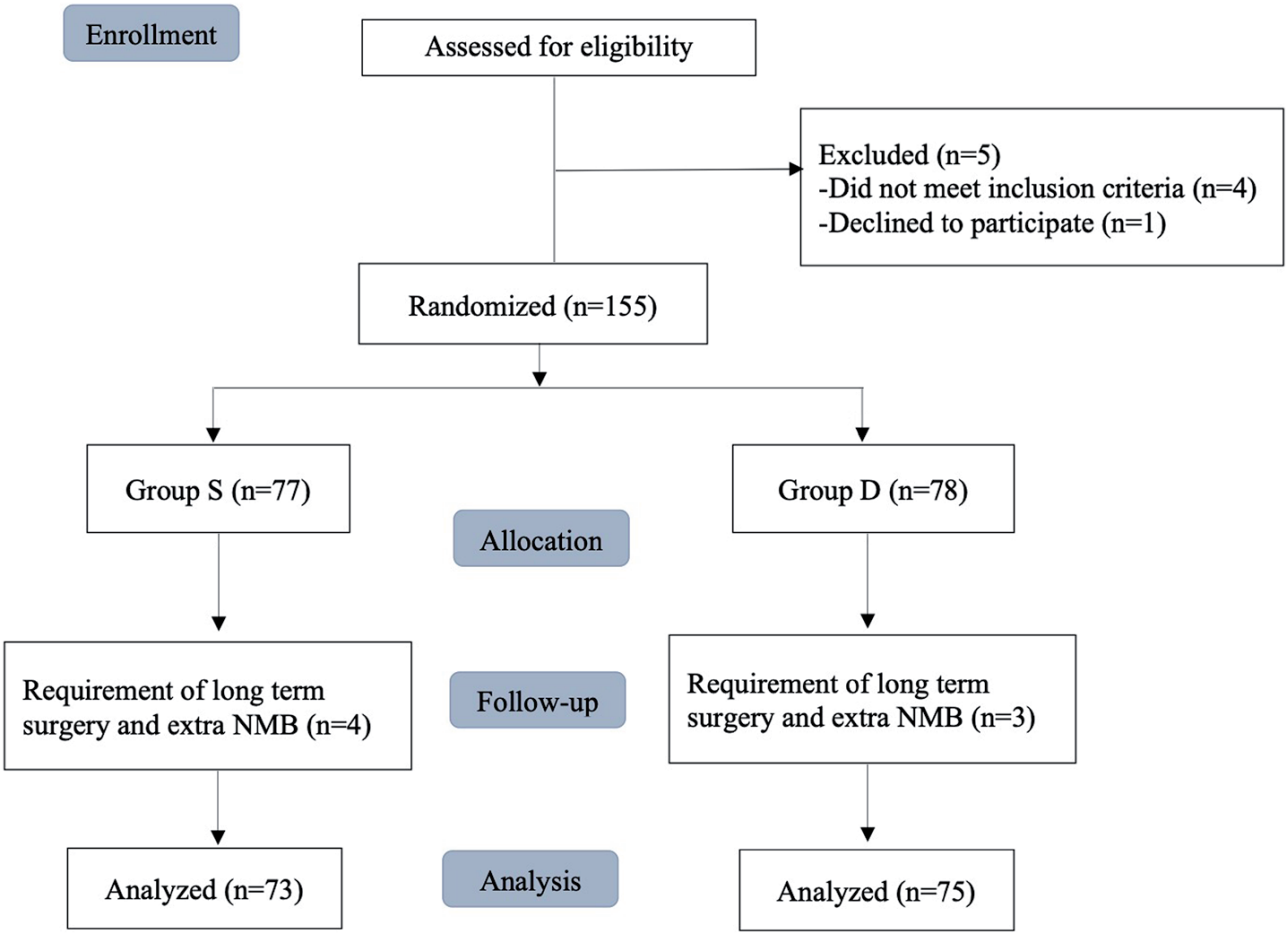
Consort flow diagram of the study.

The study enrolled 160 patients, but 7 patients were excluded from the study on grounds of the need to prolong the surgery and to use extra dose of neuromuscular blocker, 1 patient was excluded after refusal by the parents to give consent for inclusion, and 4 patients that did not meet inclusion criteria were excluded from study (Figure 1).

A total of 148 patients comprising 114 males (77%) and 34 females (23%) were included in the study (Figure 1 and Table 1). The group S and group D patients and their respective age subgroups did not significantly differ with respect to demographic data or the type of surgery performed (Table 1).

The mean *T*_S_ value in group S was significantly lower than in group D (*P* =.03). Similarly significant differences were not observed in the *T*_S_ values of the 5-10-year-old subgroup of patients in group S and group D (Table 2).

No statistically significant differences were observed between PIP values in groups S and D at all time points (Table 3). When the PIP values within each group were compared, before and 1, 2, 5, and 10 minutes after sugammadex injection, the PIP values at 2, 5, and 10 minutes after sugammadex injection were significantly higher in the group D (*P* values .02, .003, .006, .006, respectively; Bonferroni correction *P* < .017) as compared to the 0 minute value, while no significant differences were observed in PIP values for group S (*P* > .05).

The mean values of SAP-DAP measurements of the 2 groups 0, 1, 2, 5, and 10 minutes after sugammadex injection did not differ significantly (*P* > .05). Bradycardia was observed in 2 male patients, 4 and 9 years of age placed under sevoflurane anesthesia with HR dropping, respectively, from 100 to 86 and from 125 to 100, and in 1 male patient, 9 years of age placed under desflurane anesthesia with HR dropping from 74 to 62. Side effects such as rashes or recurarisation were not observed in any of the patients in the recovery room.

## Discussion

The development of the neuromuscular junction is completed by 2 years of age, individual variations are possible such that some studies have demonstrated the smaller and shorter postsynaptic area and membrane length in 2-month-old babies and children up to the age of 4 years when compared to adults.^1^ Because of this situation, there is always the possibility of these differences resulting in dramatic changes in the neuromuscular blockade and its reversal even within small age intervals. Therefore, the patients were investigated by forming subgroups of 2-4 and 5-10 years of age.

The primary target of our study was to investigate the 2-10-year-old patients. *T*S is the time taken for TOF 0.25 to reach at least 0.90 after sugammadex injection. The TOF > 0.90 was reached in less than a mean duration of 1 minute in both groups. Also, group S and group D did not differ with respect to the speed of block reversal (Table 2).

Investigating the possibility of an age-dependent difference of the sugammadex effect, it was found that the reversal of the rocuronium blockade was faster with results of mean 45.6 ± 18.5 seconds under sevoflurane anesthesia as compared to 56.8 ± 19.7 seconds under desflurane anesthesia in the 2-4-year-old subgroup, but a similar significant *T*_S_ difference was not reached in the 5-10-year-old subgroups of groups S and group D. However, the time of sugammadex reversal effect did not differ between the 2 age subgroups in group S and in group D taken separately (Table 2).

Düger et al^3^ investigated in adults the possibility of different effects of MAC 1.25 isoflurane, desflurane, and sevoflurane anesthesia on sugammadex reversal of neuromuscular block by 0.6 mg/kg rocuronium with 0.15 mg/kg maintenance dose added when TOF had reached 0.25. The time taken (*T*_S_) for TOF 0.25 to reach 0.90 after 2 mg/kg sugammadex injection at the termination of surgery was found to be shorter under sevoflurane anesthesia. Although this study technically differed from ours with respect to using more than a single dose of rocuronium, the MAC adjustment, and working with adults, we also observed a similar difference between sevoflurane and desflurane anesthesia in the 2-4-year-old subgroup of patients.

In an investigation with limited numbers of infant, child, adolescent, and adult patients, sugammadex reversal of the single 0.6 mg/kg dose of rocuronium blockade was found to be faster in infants as compared to adults, irrespective of the inhaled anesthetic agents.^4^ The time taken to reach TOF 0.90 after sugammadex injection to reverse deep rocuronium block was reported to be 1.3 minutes in the 20-50-year-old patients as compared to 3.6 minutes in the elderly patients **> **70 years of age.^5^ According to Matsui et al,^2^ the duration of the reversal is inversely proportional to the cardiac output. The faster increase in sugammadex concentration in the neuromuscular junction results in faster recovery. As age decreases, the block reversal therefore gets faster, as also shown in our study by the very fast reversal of the blockade in the 2-10-year-old patients, although a difference in the rate of recovery was not found between the 2 subgroups of patients in this study with very small age difference (2-4 and 5-10 years old).

A review of 10 studies on a total of 580 pediatric patients by taking TOF > 0.9 as the reference parameter demonstrated that sugammadex reversed rocuronium blockade faster and with less incidences of bradycardia as compared to placebo and neostigmine.^6^

There are also case reports discussing increased sugammadex injection leading to hypersensitivity and bronchospasm. In a review on sugammadex-related hypersensitivity, attention was drawn to the necessity of awareness on the side effects of this agent especially during the most critical period of 5 minutes following its administration.^7^ With the increased use of sugammadex, case reports especially on incidences of bronchospasm and anaphylactic hypersensitivity are being reported in the literature.^8,9^

Similarly, incidences of bronchospasm after sugammadex injection during desflurane anesthesia have also been reported.^10,11^ The impact mechanism of sugammadex does not involve interaction with the cholinergic system. It has also been shown not to affect the contractility of the bronchial smooth muscle of the rat.^12^

In our study, we adjusted the inhaled agents to 1 MAC, and routinely monitored the PIP before (i.e., at 0 time) and during the 10 minutes after sugammadex injection. Although there were not any differences in the PIP values at 0, 1, 2, 5, and 10 minutes of the 2-10-year-old patients in group S, they were higher during the 1-10 minutes as compared to the 0 time level in group D. However, the observed increase from the 0 time control level of 15.6 ± 2.8 cmH_2_O to the highest level of 16.0 ± 2.9 cmH_2_O level does not have clinical significance (Table 3). Previous studies showed that 1.5 MAC desflurane anesthesia might cause increased airway pressure; however, it was not repeated under 1 MAC adjustment.^13^ Conclusions on whether desflurane increases airway irritation and resistance and whether its use with sugammadex would increase these adverse side effect incidences will require more case reports on the subject.

The results of a meta-analysis on the comparison of sugammadex with atropine and neostigmine did not show significant differences in relation to the side effects of bronchospasm and desaturation in children.^14^ However, there are case reports on the incidences of persistent bradycardia and cardiac arrest after sugammadex use in patients of different age groups. Between 2009 and 2017 in the United States, 38 cases of bradycardia, 39 cases of cardiac arrest, 10 cases of ventricular fibrillation, and 8 cases of ventricular tachycardia were seen after sugammadex use in a total of 138 adult patients.^15^ In relation to these reports, we observed in our study the SAP, DAP, and HR in our pediatric patients before and within the critical time of 10 minutes after sugammadex injection and did not obtain any statistically significant changes in the recorded data, except for three male patients who developed bradycardia at the first minute after sugammadex injection which did not require treatment. The Anaesthetic and Analgesic Drug Products Advisory Committee^16^ stated that after sugammadex use, the incidence of bradycardia was 1% and even if it results in cardiac arrest, it is dose dependent and temporary. According to another study, no side effects of sugammadex were found in 331 patients under the age of 2 years.^17^ These reports evince that sugammadex is not different from the other cholinesterase inhibitors with respect to side effects and that it is a fast-acting and reliable agent in reversing the neuromuscular blockade by the 0.6 mg/kg single dose of rocuronium in patients of different age groups.^4^

In a very recent investigation of 221 children under the age of 18 years, bradycardia was observed at a median of 2 minutes time after sugammadex injection in 18 patients, of whom 7 had comorbid congenital heart disease. Multivariate regression analysis showed that the incidence of bradycardia, next to cardiac comorbidity, was more prevalent among the males. Blood pressure was stable and there was no occurrence of clinical side effects requiring treatment.^18^

In another study with patients under the age of 18 years, the incidence of bradycardia was rare even in the presence of congenital heart disease and was seen more frequently in males. Males with older age or higher BMI were more vulnerable to bradycardia but this situation did not cause hemodynamic impairment and did not require treatment.^19^ These results, as well as ours, are encouraging for the use of sugammadex as an agent with low incidences of bradycardia and negative impact on hemodynamics.

This study also suffers from a few limitations. First, not including an internal control group of patients with anticholinesterase use instead of sugammadex can be cited as one. Second limitation is that we did not administer different doses of sugammadex in our study. The sugammadex dosage can be changed from the TOF assessment. We chose the 2 mg/kg dose because we administered sugammadex when the TOF value reached 0.25. Another limitation may be the exclusion of infants under the age of 2 years in the developmental stage of the neuromuscular junction and the inclusion of children with very close age intervals.

In this study, we show that administering a 2-mg/kg dose of sugammadex when the neuromuscular block is induced by a single dose of 0.6 mg/kg rocuronium while TOF is 0.25 resulted in very rapid and effective recovery in a total of 148 children of 2-10 years of age operated under sevoflurane or desflurane anesthesia. Reversal of the block was significantly faster in the 2-4-year-old subgroup as compared to the 5-10-year-old subgroup. In group D patients, clinically insignificant 1 cmH_2_O increase in PIP was observed after sugammadex injection. There were no hemodynamic changes in SAP, DAP, and HR, and only 3 cases of bradycardia were seen in male patients of 9 (n = 2) and 4 years of age.

In our study, no adverse side effects such as bronchospasms, anaphylactic hypersensitivity occurred and other complications or recurarization were not observed in the recovery room.

We believe that further research is needed about the use of 2 mg/kg dose of sugammadex to reverse neuromuscular block induced by a single dose of 0.6 mg/kg rocuronium in order to confirm sugammadex’s rapidity and effectiveness of recovery and also to determine its side effects and the safety of sugammadex use in combination with other agents.

## Figures and Tables

**Table 1.** Comparison of the Demographic and Clinical Data of the Patients

	Group S		Group D		*P*
Gender (n, %)					
Male	58	79.5	56	74.7	.49
Female	15	20.5	19	25.3	
Age mean ± SD (min-max)	5.3 ± 2.8 (2-10)		5.4 ± 3.0 (2-10)		.89
2-4 years (n, %)	35	47.9	37	49.3	.87
5-10 years (n, %)	38	52.1	38	50.7	
Weight (kg) mean ± SD (min-max)	23.2 ± 12.7 (8-62)		23.6 ± 13.5 (8-63)		.99
Height (cm) mean ± SD (min-max)	134.6 ± 32.1 (88-187)		124.9 ± 27.0 (85-180)		.38
BMI mean ± SD (min-max)	17.5 ± 3.1 (12.4-22.2)		18.0 ± 4.3 (9.2-25.2)		80
Surgical procedures (n, %)					
Cystoscopy	35	47.9	43	57.3	.51
Lower abdominal surgery	30	41.0	26	34.7	
Examination under anesthesia	8	11.0	6	8.0	

Data are presented as mean ± standard deviation (SD), minimum-maximum, n: number, percentages (%).

BMI, body mass index.

**Table 2.** Comparison of the Sugammadex Effect

*T*_S_ (s)	2-4 years	5-10 years	2-10 years
Group S Mean ± SD	n = 35	n = 38	n = 75
	45.6 ± 18.5	55.6 ± 26.5	50.8 ± 23.4
Group D Mean ± SD	n = 37	n = 38	n = 75
	56.8 ± 19.7	59.4 ± 27.2	57.2 ± 24.4
*P*	.03	.67	.11

Data are presented as mean ± standard deviation (SD). n, number; y, years old.

*T*_S_ = time taken for TOF value to reach from 0.25 to minimally 0.90.

**Table 3.** Comparison of the PIP Values between the Groups

PIP (mean ± SD)	Group S	Group D	*P*
0 min	15.5 ± 2.5	15.6 ± 2.8	.63
1 minute	15.7 ± 2.4	15.9 ± 2.8	.62
2 minutes	15.8 ± 2.5	15.9 ± 2.9	.74
5 minutes	15.8 ± 2.4	16.0 ± 2.9	.68
10 minutes	15.8 ± 2.5	16.0 ± 2.9	.77

Data are presented as mean ± standard deviation (SD). PIP, peak inspiratory pressure.
